# Diabetes and other vascular risk factors in association with the risk of lower extremity amputation in chronic limb-threatening ischemia: a prospective cohort study

**DOI:** 10.1186/s12933-021-01441-0

**Published:** 2022-01-08

**Authors:** Ariel Fangting Ying, Tjun Yip Tang, Aizhen Jin, Tze Tec Chong, Derek John Hausenloy, Woon-Puay Koh

**Affiliations:** 1 Cardiovascular and Metabolic Disorders Program, Duke-National University of Singapore Medical School, Singapore, Singapore; 2 Department of Vascular Surgery, Singapore General Hospital, Singapore, Singapore; 3 Healthy Longevity Translational Research Programme, Yong Loo Lin School of Medicine, National University of Singapore, 5 Science Drive 2, Singapore, 117545 Singapore; 4 National Heart Research Institute Singapore, National Heart Centre, Singapore, Singapore; 5 Yong Loo Lin School of Medicine, National University Singapore, Singapore, Singapore; 6 The Hatter Cardiovascular Institute, University College London, London, UK; 7 Cardiovascular Research Center, College of Medical and Health Sciences, Asia University, Taichung, Taiwan; 8 Singapore Institute for Clinical Sciences, Agency for Science Technology and Research (A*STAR), Singapore, Singapore

**Keywords:** Diabetes and peripheral vascular disease, Clinical epidemiology, Diabetes and lower limb amputation, Cardiovascular risk factors, Vascular epidemiology

## Abstract

**Background:**

Patients with diabetes are at increased risk of developing chronic limb-threatening ischemia (CLTI) due to peripheral arterial disease, and this often results in lower extremity amputation (LEA). Little is known of the interaction between diabetes and other vascular risk factors in affecting the risk of CLTI.

**Methods:**

We investigated the association of diabetes, and its interaction with hypertension, body mass index (BMI) and smoking, with the risk of LEA due to CLTI in the population-based Singapore Chinese Health Study. Participants were interviewed at recruitment (1993–1998) and 656 incident LEA cases were identified via linkage with nationwide hospital database through 2017. Multivariate-adjusted Cox proportional hazards models were used to compute hazard ratios (HRs) and 95% CIs for the associations.

**Results:**

The HR (95% CI) for LEA risk was 13.41 (11.38–15.79) in participants with diabetes compared to their counterparts without diabetes, and the risk increased in a stepwise manner with duration of diabetes (*P* for trend < 0.0001). Hypertension and increased BMI independently increased LEA risk in those without diabetes but did not increase the risk in those with diabetes (*P* for interaction with diabetes ≤ 0.0006). Conversely, current smoking conferred a risk increment of about 40% regardless of diabetes status.

**Conclusions:**

Although diabetes conferred more than tenfold increase in risk of LEA, hypertension and increased BMI did not further increase LEA risk among those with diabetes, suggesting a common mechanistic pathway for these risk factors. In contrast, smoking may act via an alternative pathway and thus confer additional risk regardless of diabetes status.

## Introduction

Peripheral arterial disease (PAD) is an atherosclerotic occlusive disease of the lower extremity arteries and afflicts hundreds of millions of individuals worldwide [1]. Its most severe manifestation is chronic limb-threatening ischemia (CLTI), which is defined by the presence of ischemic rest pain, lower limb ulceration, or gangrene for more than 2 weeks in duration [2, 3]. Despite the significant risk of impaired quality of life, physical disability, and even mortality, most studies have focused on less severe forms of PAD, such as those diagnosed by low ankle-brachial index or history of intermittent claudication. Indeed, both the 2016 American Heart Association guidelines for PAD [4] and the 2019 global vascular guidelines for CLTI [2] have identified a dearth of evidence on the epidemiology of more severe forms of PAD, and highlighted the critical need for further research in this area.

It is becoming increasingly recognized that those with diabetes have a different clinical profile when it comes to PAD [5]. Compared to stable forms of PAD such as intermittent claudication or subclinical disease detected by low ankle-brachial index, diabetes is more strongly associated with severe manifestations like CLTI [6–8], and patients with diabetes frequently present with de novo tissue loss and without prior diagnosis of PAD or symptoms of intermittent claudication [9, 10]. In epidemiological studies that examined the impact of diabetes on the risk of developing CLTI in individuals with no known history of PAD, multivariate-adjusted risk ratios as high as 5.96 (95% CI 3.15–11.26) and 7.45 (95% CI 7.19–7.72) have been reported [7, 11]. In addition, those with diabetes are up to five times as likely to require a major lower extremity amputation (LEA), possibly due to higher risk of infection, as well as a higher likelihood of having disease in the distal arteries, thus rendering revascularization technically challenging [9, 12].

Despite this significant difference in morbidity, most epidemiological studies examining associations between diabetes and PAD risk have included in their assessments stable forms of PAD that may be managed conservatively, while studies on the severe forms of PAD requiring surgical intervention have been largely limited to either retrospective data or small-sized analyses of pre-selected patient populations [13], and the few prospective studies in this field did not focus exclusively on LEA [11]. To the best of our knowledge, there has been no large prospective cohort study that directly examined the impact of diabetes and its interaction with other vascular risk factors on the risk of developing CLTI-associated LEA [14].

Singapore has one of the highest rates of diabetes (14.2%) [15] as well as LEA rates for CLTI [16] in the world. Thus, the main aim of this study nested in a Singapore cohort was to prospectively assess the associations between a baseline history of diabetes and the risk of developing CLTI-associated LEA. We also aimed to understand how diabetes may interact with other vascular risk factors, which frequently co-exist with diabetes, to modify this risk. Data was obtained from the Singapore Chinese Health Study [17], a large population-based prospective cohort of Chinese men and women followed up over a mean of 18.8 years.

## Research design and methods

### Study population

This study is nested in the Singapore Chinese Health Study cohort, which has been previously described in detail [17]. In summary, this is a population-based cohort that was established to study dietary, genetic, and environmental determinants of cancer and other chronic diseases in Singapore. A total of 63,257 middle-aged or older participants (27,954 men and 35,303 women) between the ages of 45 and 74 years were enrolled prospectively over a 5-year period (April 1993 to December 1998). They were drawn from a pool of citizens and permanent residents from Singapore who resided in government purpose-built housing estates, where 86% of the entire Singapore population lived at the time of recruitment. The Institutional Review Board at the National University of Singapore had approved this study and written informed consent was obtained from each subject.

### Assessment of diabetes and other vascular risk factors

At recruitment, trained interviewers conducted in-person interviews with each subject at his or her home. Self-reported information about age, weight, height, usual diet, tobacco use, physical activity, level of education and alcohol intake was obtained using a structured questionnaire. Body mass index (BMI) was calculated as body weight in kilograms divided by square of height in meters. Smokers were classified as never smokers, former smokers, and current smokers. Alcohol consumption was classified as “never”, “at least once a month but less than once a week”, “at least once a week but less than daily”, and “daily”. The participants were also asked whether they had a history of physician-diagnosed medical conditions, including diabetes and hypertension, and if yes, the number of years they have had the condition. Using standard protocols, we had conducted separate studies to validate the accuracy of the self-reported, physician-diagnosed diabetes [18] and hypertension [19] in this cohort, and they had been reported previously.

### Diagnosis of CLTI

We first identified LEA cases as participants in this cohort who had undergone any amputation (major, minor, or digital) in the lower limb for CLTI through record linkage of cohort files with databases of the Singaporean MediClaim System, which has captured all inpatient discharge information from all private and government restructured hospitals in Singapore since 1990. We only recorded the first instance of LEA for each case. All LEA cases were verified by records of the appropriate surgical procedural codes, and we excluded LEA due to non-vascular causes using International Classification of Disease Version 9 (ICD-9) and Version 10 (ICD-10) codes for trauma (ICD-9 800-999; ICD-10 S00-Y99), peripheral neuropathy (ICD-9 355, 356, 357; ICD-10 G57, G60-G65), cancers of the bone, cartilage, and nerves (ICD-9 170.6-170.9, 171.3, 171.6-171.9; ICD-10 C40.2-C40.9, C47.2, C47.5-C47.9), necrotizing fasciitis (ICD-9 728.86), and osteomyelitis or osteonecrosis (ICD-9 730; ICD-10 M86-M87) not due to PAD.

Information on date of death was obtained through linkage analysis with the nationwide Registry of Births and Deaths. As of 31 December 2017, when vital status for cohort participants was updated, only 41 participants (0.06%) from this cohort were known to be lost to follow-up due to migration out of Singapore or for other reasons. This suggested that in this cohort, loss to follow-up was negligible and that vital statistics were virtually complete in this cohort.

### Statistical analysis

We identified 123 participants who had undergone LEA for CLTI before enrolment into the cohort and excluded them from the study. Hence, the analyses in this study were limited to the 63,134 participants without history of CTLI-related LEA at enrolment. We examined the difference in distribution of baseline characteristics between cases and non-cases, and between participants with diabetes and those without diabetes using chi-square test for categorical variables and Student’s *t*-test for continuous variables. For each study subject, person-years were counted from the date of baseline interview to the date of LEA for cases, or to the date of death, loss to follow-up or 31 December 2017 for non-cases, whichever occurred first. Cox proportional hazards regression models were used to explore the relationship between vascular risk factors and the risk of LEA due to CLTI within the entire cohort. The magnitude of the associations was assessed by hazard ratios (HR) with their corresponding 95% confidence intervals (CI).

Survival analysis was performed to generate the Kaplan–Meier curves for LEA due to CLTI, and log-rank test was used to compare the curves of those with diabetes versus those without diabetes. All models were adjusted for age at recruitment (year), year of study enrolment (1993–1995, 1996–1998), sex, dialect group (Hokkien or Cantonese), education level (none, primary school, secondary school or higher), and physical activity (<0.5 hr/wk, 0.5 to <4 hr/wk, 4+ hr/wk). Model 2 included the addition of other vascular risk factors, namely increased BMI (BMI < 23 kg/m^2^, BMI ≥ 23 kg/m^2^), smoking (never, former, current), alcohol consumption (never/monthly, weekly/daily), history of hypertension, history of coronary artery disease, and history of stroke. We also investigated the associations between other vascular risk factors, namely hypertension, increased BMI and smoking, and risk of LEA due to CLTI in the whole cohort, and in analyses stratified by history of diabetes. We tested the statistical significance of the interactions of diabetes with hypertension, BMI, smoking, and alcohol consumption on the risk of LEA from CLTI by including their respective product terms as covariates in the models.

Statistical computing was performed using SAS version 9.1 (SAS Institute Inc., Cary, NC, US). All cited p values were two-sided in nature and a *p*-value < 0.05 was considered statistically significant.

## Results

A total of 63,134 subjects, accounting for 1,189,930 person-years, were included in this analysis (Table 1). During a mean follow-up period of 18.8 (± 6.2) years, there were 656 incident LEA cases due to CLTI. Cases were older and less educated at baseline, had lower rates of physical activity per week, and were more likely to be former or current smokers. They were also more likely to have a history of diabetes, hypertension, coronary artery disease, or stroke. A total of 5616 (8.9%) participants reported a history of physician-diagnosed diabetes at baseline. They were also more likely to have a history of hypertension, coronary artery disease, or stroke than their counterparts without a history of diabetes.

**Table 1:** Baseline characteristics of cohort by lower extremity amputation (LEA) status and diabetes status

	LEA cases	Non-cases	Diabetes	No diabetes
Number of subjects	656 (1.0%)	62,478 (99.0%)	5616 (8.9%)	57,518 (91.1%)
Men	318 (48.5%)	27,562 (44.1%)	2374 (42.3%)	25,506 (44.3%)
Women	338 (51.5%)	34,916 (55.9%)	3242 (57.7%)	32,012 (55.7%)
Age (year), mean (SD)	59.4 (7.9)	56.5 (8.0)	60.0 (7.7)	56.2 (8.0)
Dialect				
Hokkien	256 (39.0%)	28,983 (46.4%)	2588 (46.1%)	26,651 (46.3%)
Cantonese	400 (61.0%)	33,495 (53.6%)	3028 (53.9%)	30,867 (53.7%)
Level of education				
No formal education	267 (40.7%)	17,028 (27.2%)	1980 (35.3%)	15,315 (26.6%)
Primary school	271 (41.3%)	27,713 (44.4%)	2461 (43.8%)	25,523 (44.4%)
Secondary or higher	118 (18.0%)	17,737 (29.4%)	1175 (20.9%)	16,680 (29.0%)
Physical activity				
< 0.5 h/wk	466 (71.0%)	41,910 (67.1%)	3928 (69.9%)	38,448 (66.8%)
0.5 to < 4 h/wk	108 (16.5%)	12,644 (20.2%)	1030 (18.4%)	11,722 (20.4%)
≥ 4 h/wk	82 (12.5%)	7924 (12.7%)	658 (11.7%)	7348 (12.8%)
BMI (kg/m^2^), mean (SD)	23.9 (3.3)	23.1 (3.3)	24.0 (3.3)	23.0 (3.3)
Smoking				
Never	408 (62.2%)	43,463 (69.6%)	3839 (68.4%)	40,032 (69.6%)
Former	90 (13.7%)	6873 (11.0%)	884 (15.7%)	6079 (10.6%)
Current	158 (24.1%)	12,142 (19.4%)	893 (15.9%)	11,407 (19.8%)
Alcohol drinking				
Never/monthly	584 (89.0%)	55,244 (88.4%)	5233 (93.2%)	50,595 (88.0%)
Weekly/daily	72 (11.0%)	7234 (11.6%)	383 (6.8%)	6923 (12.0%)
Medical history				
Diabetes	346 (52.7%)	5270 (8.4%)	–	–
Hypertension	225 (34.3%)	14,782 (23.7%)	2663 (47.4%)	12,344 (21.5%)
Coronary artery disease	53 (8.1%)	2528 (4.1%)	688 (12.3%)	1893 (3.3%)
Stroke	31 (4.7%)	909 (1.5%)	268 (4.8%)	672 (1.2%)

*P*-value < 0.05 for all except dialect in diabetes by Student’s t-test or ANOVA

**Fig. 1:**
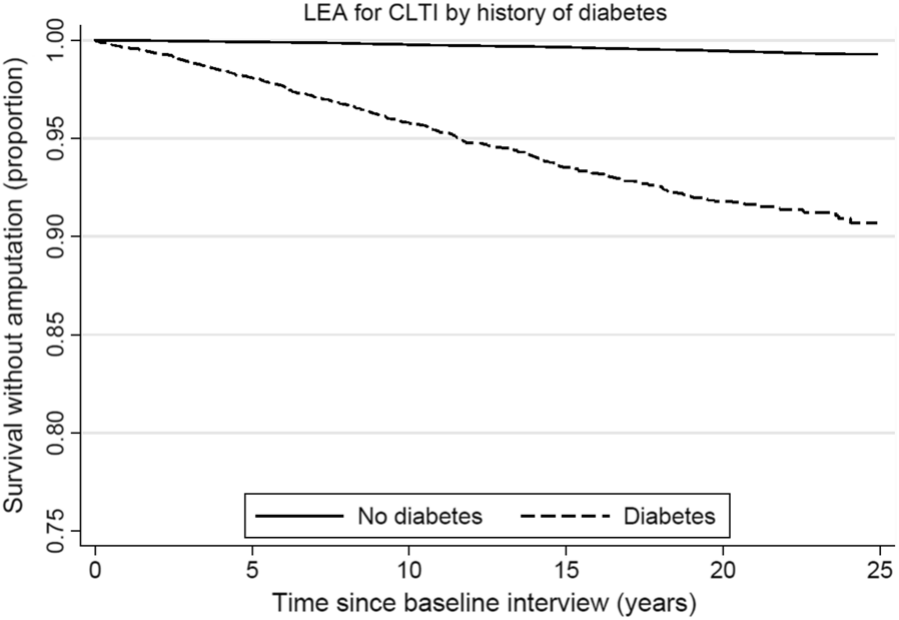
Kaplan–Meier survival curves of lower extremity amputation (LEA) for chronic limb-threatening ischemia (CLTI) by history of diabetes

In Fig. 1, Kaplan–Meier survival curves showed that participants with diabetes had significantly lower probability of LEA than those without diabetes over time. In multivariable Cox models, compared to those without diabetes, those with diabetes had a 13-fold increase in the risk of LEA for CLTI (HR, 13.61; 95% CI 11.64–15.91), which was not materially attenuated after adjusting for other vascular risk factors (HR, 13.41; 95% CI 11.38–15.79). The risk of LEA for CLTI was also found to increase in a stepwise manner with duration of diabetes (*p* for trend < 0.0001). Compared to those without diabetes, those with diabetes for at least 15 years had a 23-fold increase in the risk of LEA for CLTI (HR, 23.22; 95% CI 18.16–29.67). There was no significant difference in risk estimates between men and women (Table 2).

**Table 2:** Association between diabetes and the risk of lower extremity amputation for chronic limb-threatening ischemia: Cox proportional hazards regression analysis

Characteristics	Person-years	Cases	HR (95% CI)^a^	HR (95% CI)^b^
All subjects				
No diabetes	1,106,992	310	1.00	1.00
Diabetes	82,938	346	13.61 (11.64–15.91)	13.41 (11.38–15.79)
Diabetes < 5 years	35,685	96	9.12 (7.25–11.48)	8.95 (7.07–11.32)
Diabetes 5 to < 10 years	21,057	71	11.33 (8.74–14.69)	11.13 (8.55–14.49)
Diabetes 10− < 15 years	14,143	88	19.98 (15.71–25.42)	19.66 (15.38–25.12)
Diabetes 15+ years	12,053	91	23.67 (18.6–30.13)	23.22 (18.16–29.67)
*P* for trend			< 0.0001	< 0.0001
Men				
No diabetes	464,606	162	1.00	1.00
Diabetes	32,863	156	13.78 (11.03–17.23)	13.78 (10.91–17.44)
Diabetes < 5 years	13,933	41	8.50 (6.03–11.98)	8.57 (6.03–12.18)
Diabetes 5 to < 10 years	8383	33	11.96 (8.22–17.41)	11.96 (8.15–17.55)
Diabetes 10− < 15 years	5517	39	20.65 (14.48–29.44)	20.22 (14.08–29.04)
Diabetes 15+ years	5030	43	25.82 (18.26–36.53)	25.58 (17.94–36.47)
*P* for trend			< 0.0001	< 0.0001
Women				
No diabetes	642,386	148	1.00	1.00
Diabetes	50,075	190	13.14 (10.54–16.38)	12.58 (9.96–15.77)
Diabetes < 5 years	21,752	55	9.45 (6.92–12.91)	8.92 (6.48–12.28)
Diabetes 5 to < 10 years	12,675	38	10.52 (7.34–15.07)	10.02 (6.96–14.43)
Diabetes 10− < 15 years	8626	49	19.00 (13.67–25.42)	18.25 (13.05–25.52)
Diabetes 15+ years	7022	48	21.48 (15.35–30.06)	20.23 (14.37–28.48)
*P* for trend			< 0.0001	< 0.0001

^a^Cox Model 1: Hazard ratio (HR) adjusted for age at recruitment, year of study enrolment, sex, dialect group, educational level, physical activity

^b^Cox Model 2: Model 1 plus body mass index, smoking, alcohol consumption, history of hypertension, history of coronary artery disease, history of stroke

We also examined the association of other traditional cardiovascular risk factors, namely hypertension, BMI, smoking status, and alcohol consumption, with the risk of CLTI-associated LEA in the whole cohort, as well as in stratified analysis by the status of diabetes (Table 3). Although hypertension and increased BMI (≥ 23.0 kg/m^2^) were risk factors in the model that did not include diabetes as a covariate, the risk estimates of both factors were attenuated and no longer reaching statistical significance after adjusting for diabetes. Further, in participants without diabetes, hypertension remained a significant risk factor for CLTI-associated LEA (HR, 1.34; 95% CI 1.04–1.73), but in those with diabetes, hypertension did not further increase this risk (HR, 0.79; 95% CI 0.63–1.00). Similarly, in participants without diabetes, BMI ≥ 23 kg/m^2^ remained a significant risk factor for CLTI-associated LEA (HR, 1.58; 95% CI 1.25–1.99), but in participants with diabetes, BMI ≥ 23 kg/m^2^ did not further increase this risk (HR, 0.89; 95% CI 0.71–1.11). Hence, as expected, there was significant interaction between diabetes and hypertension (P for interaction = 0.0006), as well as between diabetes and increased BMI (P for interaction = 0.0002), since diabetes status significantly modified the associations between these vascular risk factors and the risk of CLTI-related LEA.

**Table 3:** Association between cardiovascular risk factors and risk of lower extremity amputation for chronic limb-threatening ischemia stratified by history of diabetes: Cox proportional hazards regression analysis

	All subjects			No diabetes		Diabetes		*P* for interaction
	Cases	HR (95% CI)^a^	HR (95% CI)^b^	Cases	HR (95% CI)^a^	Cases	HR (95% CI)^b^	
Hypertension								0.0006
No hypertension	431	1.00	1.00	226	1.00	205	1.00	
Hypertension	225	1.45 (1.22–1.72)	0.95 (0.79–1.13)	84	1.34 (1.04–1.73)	141	0.79 (0.63–1.00)	
Body mass index								0.0002
< 23.0 kg/m^2^	250	1.00	1.00	118	1.00	132	1.00	
≥ 23.0 kg/m^2^	406	1.38 (1.17–1.62)	1.17 (0.99–1.37)	192	1.58 (1.25–1.99)	214	0.89 (0.71–1.11)	
Smoking								0.84
Never smoker	408	1.00	1.00	189	1.00	219	1.00	
Former smoker	90	1.15 (0.89–1.49)	1.07 (0.83–1.38)	33	0.93 (0.62–1.39)	57	1.16 (0.84–1.62)	
Current smoker	158	1.40 (1.13–1.73)	1.46 (1.19–1.80)	88	1.51 (1.12–2.03)	70	1.42 (1.06–1.91)	
Alcohol								0.88
Never/monthly	584	1.00	1.00	267	1.00	317	1.00	
Weekly/daily	72	0.96 (0.74–1.24)	1.08 (0.84–1.40)	43	1.11 (0.79–1.55)	29	1.06 (0.72–1.58)	

^a^Cox Model 2: Hazard ratio (HR) adjusted for age at recruitment, year of study enrolment, sex, dialect group, educational level, physical activity, body mass index, smoking, alcohol consumption, history of hypertension, history of coronary artery disease, history of stroke

^b^Cox Model 3: Model 2 plus history of diabetes

Conversely, current smoking was a risk factor for CLTI-associated LEA, and the hazard ratio for the association remained materially unchanged in the models with and without including diabetes as a covariate. Compared to never smokers, current smokers had an increased risk of LEA for CLTI (HR 1.40; 95% CI 1.13–1.73), and this risk was materially unchanged after adjusting for history of diabetes (HR 1.46; 95% CI 1.19–1.80). Regular alcohol consumption was not associated with LEA for CLTI risk in this cohort (HR 0.96; 95% CI 0.74–1.24) (Table 3).

We further assessed the joint effects of diabetes and other vascular risk factors found to be significant in our cohort via two-by-two tables (Table 4). First, using participants with no diabetes and hypertension as the referent, those with both diabetes and hypertension did not have higher risk than those who only had diabetes. Similarly, using participants who had neither diabetes nor increased BMI as referent, those with both risk factors also did not have higher risk than those who only had diabetes. In contrast, compared to those who were not smoking and had no history of diabetes, participants who smoked and also had diabetes had the highest risk estimate (HR 19.12; 95% CI 14.47–25.26) compared to those who only had one risk factor, and this HR approximated to the expected risk estimate (HR 19.37) expected from the joint effects of diabetes and smoking in a multiplicative model.

**Table 4:** Joint effects between cardiovascular risk factors and diabetes for risk of lower extremity amputation for chronic limb-threatening ischemia via two-by-two table: Cox proportional hazards regression analysis

	No diabetes		Diabetes	
	Cases	HR (95% CI)	Cases	HR (95% CI)
Hypertension				
No hypertension	226	1.00	205	16.10 (13.27–19.53)
Hypertension	84	1.34 (1.04–1.73)	141	12.07 (9.62–15.16)
Body mass index				
< 23.0 kg/m^2^	118	1.00	132	19.33 (14.98–24.95)
≥ 23.0 kg/m^2^	192	1.58 (1.25–1.99)	214	16.92 (13.36–21.43)
Smoking				
Never or former smoker	222	1.00	276	13.45 (11.17–16.19)
Current smoker	88	1.44 (1.11–1.87)	70	19.12 (14.47–25.26)

Cox Model: Hazard ratio (HR) adjusted for age at recruitment, year of study enrolment, sex, dialect group, educational level, physical activity, body mass index, smoking, alcohol consumption, history of hypertension, history of coronary artery disease, history of stroke

## Discussion

In this large population-based prospective cohort, we found that diabetes conferred a more than tenfold increase in risk of LEA for CLTI, and the risk increased with duration of diabetes in a stepwise manner. Furthermore, although hypertension and increased BMI were risk factors of LEA due to CLTI amongst those without diabetes, these two cardiovascular risk factors did not further increase the risk for LEA amongst those with diabetes. Smoking increased the risk of severe CLTI to a similar extent in both groups of participants, and diabetes did not modify the smoking-CLTI risk association.

To our best knowledge, this is the first prospective cohort study focused exclusively on LEA resulting from CLTI, the most severe clinical outcome of PAD [20–22], and we have reported the highest risk estimate for the association between a history of diabetes and the risk of severe CLTI. Several studies have examined associations between diabetes and the risk of an individual presenting initially with CLTI but with no known history of PAD. Kumakura et al*.* [6] reported a multivariate-adjusted odds ratio of 1.82 (95% CI 1.07–3.08; *p*-value = 0.028) for the 185 CLTI patients treated at their hospital in Japan, while Nehler et al*.* [7] reported a much higher multivariate-adjusted odds ratio of 7.45 (95% CI 7.19–7.72) in their United States healthcare claims database involving 18,251 CLTI patients, and Howard et al*.* [11] reported an age- and sex-adjusted risk ratio of 5.96 (95% CI 3.15–11.26) for 202 CLTI patients from the Oxfordshire OXVASC cohort. Epidemiological studies have previously found stronger associations between diabetes and the risk of developing CLTI compared to the risk of less severe forms of PAD [6, 8]. This phenomenon of patients with diabetes presenting with CLTI as the first presentation of PAD can be explained by the observation that in patients with diabetes, the classic progression of PAD is frequently absent; as many as 50% of patients with diabetes do not have a preceding history of intermittent claudication, which may be due to the presence of peripheral neuropathy [10]. As such, patients with diabetes may be unaware that they have PAD until it progresses to the tissue loss stage, thus explaining the epidemiological finding that the association between diabetes and CLTI is stronger than that between diabetes and less severe forms of PAD [7]. In addition, diabetes is also associated with factors that may affect the choice of primary treatment. The pattern of occlusive disease in patients with diabetes is usually more distal in nature and involves long segments of calcification [23], which presents technical challenges for revascularization [9]. Moreover, diabetes is associated with a higher risk of complications in CLTI such as ulceration or wound infection, which further increases the risk of requiring an LEA [9].

Hyperglycemia is a well-established risk factor for the development of PAD [20], and the underlying pathogenesis is believed to be driven by the glucose-induced formation of advanced glycation end products (AGEs) [24]. These AGEs increase the uptake of oxidized low-density lipoproteins into macrophages, and the latter evolve to foam cells that accumulate in the subendothelial area of the arterial wall to form atherosclerotic lesions [25]. Furthermore, in patients with diabetes, the very high risk estimates may be explained by the fact that AGEs are excreted via the kidneys [26]. Studies have shown that one of the major microvascular complications of diabetes, particularly long-standing diabetes, is an impairment in renal function [27]. As such, patients with diabetes are not only exposed to an increased production of AGEs, but they are also unable to excrete them effectively, thus leading to accelerated accumulation of these AGEs and hence overactivation of the AGE-RAGE axis. In addition, diabetes is known to preferentially affect distal small vessels that are less amenable to surgical revascularization, which further predisposes these patients to LEA [9, 28]. Indeed, observational studies have found associations between higher AGE receptor (RAGE) levels and increased LEA risk in patients with diabetes [29], as well as higher RAGE levels in patients with diabetes-related LEA as compared to trauma-related LEA [30]. Since the treatment of CLTI is for limb salvage wherever possible [2–4], this propensity to affect distal small vessels likely explains why diabetes induces a much higher risk of LEA than the other vascular risk factors.

In the examination of other vascular risk factors, we reported, for the first time, that while hypertension and increased BMI may be risk factors of severe CLTI requiring LEA in those without diabetes, these two factors may have no further effect on LEA risk in the presence of diabetes. Studies on the etiology of PAD have previously proposed a common pathway for diabetes, hypertension, and obesity via the formation of AGEs and increased uptake of low-density lipoproteins into macrophages [31]. Hence, it is likely that these three factors share common pathways in the development of CLTI. Thus, it is biologically plausible that in the presence of diabetes, additional risk factors of hypertension and obesity are no longer conferring further risk.

Our study concurs that smoking is an independent risk factor for CLTI regardless the status of diabetes. Cigarette smoke is implicated in inflammatory processes that drive the development of atherosclerotic lesions [32, 33]. Cigarette smoke has been implicated in platelet activation and deposition in the vascular wall, eventually leading to atherosclerosis [34, 35]. As such, smoking is able to drive the development of CLTI via mechanistic pathways that are independent of diabetes, and hence smokers with diabetes concurrently will have the combined effects of both factors in raising the risk of severe CLTI.

The main strengths of this study include the large cohort size, the prospective methodology, the long follow-up period, the clinically significant endpoint, and the usage of baseline medical history as well as other lifestyle factors that were obtained before LEA. This reduces potential recall bias or reverse causation and allows us to determine a temporal sequence in the risk association between diabetes and LEA, as well as to separately examine the effect of other vascular risk factors. In addition, this study was performed in Singapore, a small city-state with easy access to specialized healthcare. To reduce bias arising from misclassification of outcome, we used a nationwide database of hospital records containing information about hospitalization from all public and private hospitals in Singapore since 1990, which allowed us to comprehensively capture the LEA cases in the cohort via linkage analysis. We also further excluded LEAs due to all possible non-vascular causes. This ensured that the identification of cases was comprehensive and valid.

We acknowledge the several limitations in our study. First, our diagnosis of diabetes was obtained through self-reports; nonetheless at least 98% of participants with diabetes had had their diabetes status verified either via record linkage or through a second interview specific to the diagnosis, treatment or complications of diabetes [18]. Second, diabetes and the other risk factors were assessed at baseline in this study and we did not consider the subsequent development of diabetes or change in status of the other risk factors. Given the prospective design of the study, this could have led to non-differential misclassification and possible attenuation of the risk estimates. Further, since the focus of our study was on severe CLTI that necessitated LEA, we did not have baseline information about CLTI or PAD that did not require surgical intervention. Hence, we could not confidently extrapolate our findings to stable CLTI or less severe forms of PAD that could be managed conservatively. Finally, we did not have data on the treatment or control of diabetes, the presence of other diabetic microvascular complications that may increase LEA risk, or the presence of potential confounders such as hyperlipidemia, C-reactive protein and homocysteine levels [36].

## Conclusions

In conclusion, in this large population-based prospective cohort in an Asian population, we found very strong associations between diabetes and CLTI-associated LEA. Our study also suggests that diabetes, hypertension and increased BMI may induce CLTI via common pathways, while smoking induces the disease via a different mechanism. Given the high risk of morbidity and mortality in CLTI patients post-LEA, a better understanding of the mechanisms that predispose patients to non-salvageable limbs is critical to developing better prevention and treatment strategies for this high-risk group.

## Data Availability

The data that support the findings of this study are available from the Ministry of Health in Singapore but restrictions apply to the availability of these data, which were used under license for the current study, and so are not publicly available. Data are however available from the authors upon reasonable request and with permission of the Ministry of Health in Singapore.

## Abbreviations

AGE: Advanced glycation end product
BMI: Body mass index
CI: Confidence interval
CLTI: Chronic limb-threatening ischemia
HR: Hazard ratio
LEA: Lower extremity amputation
PAD: Peripheral arterial disease
